# Supplementary figures and images for: A phase-shifting anterior-posterior network organizes global phase relations
Source: PLoS One. 2024 Feb 12;19(2):e0296827. doi: 10.1371/journal.pone.0296827 (PMC10861041; doi:10.1371/journal.pone.0296827)

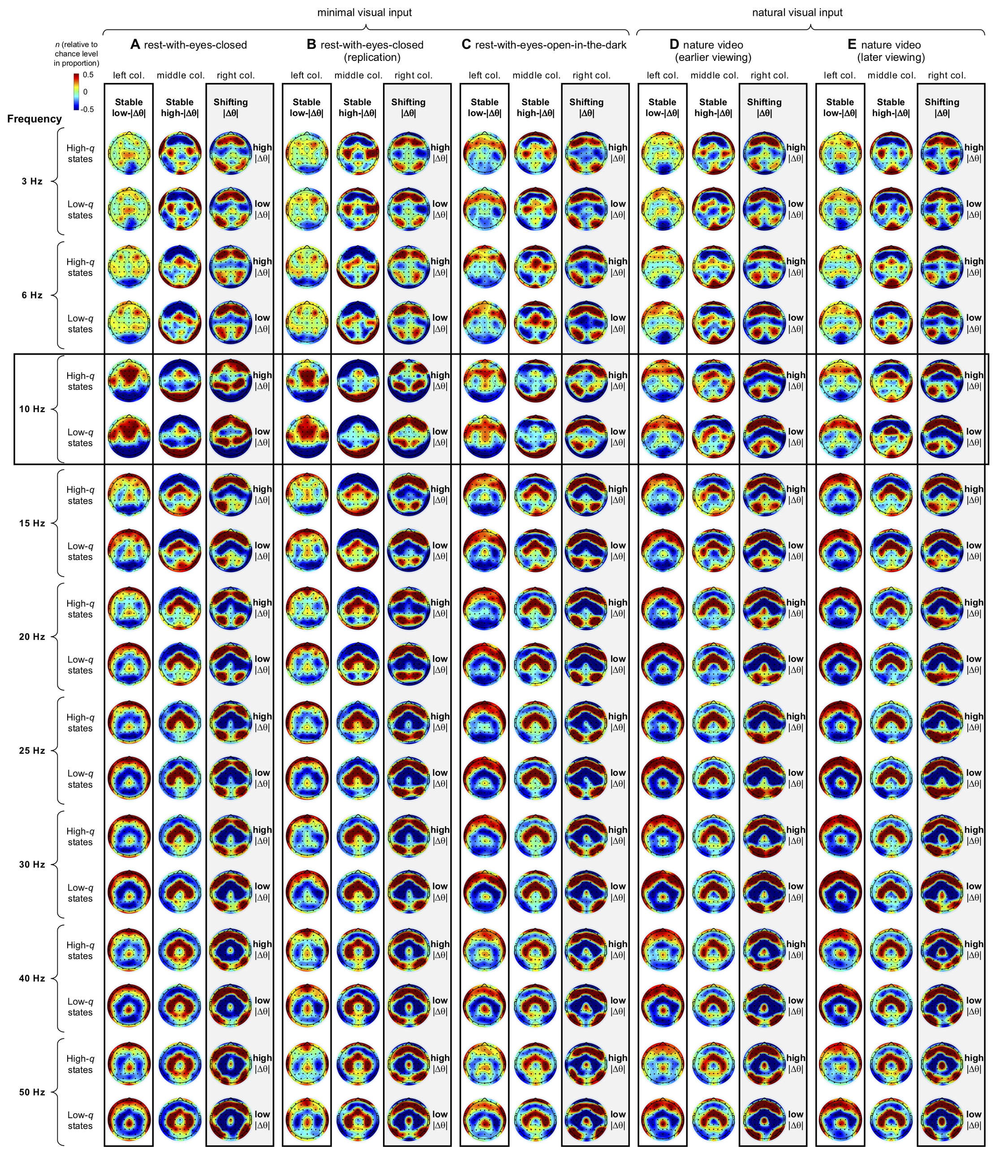

Supplement: S1 Fig — This is the same as Fig 7 except that connectivity degrees are indicated as proportions of deviations from the chance levels (rather than t-values). For instance, a value of 0.3 (or -0.3) would indicate that the corresponding connectivity degree was 30% more (or less) than expected by chance. See the color bar in the upper left corner. (TIF) [file pone.0296827.s001.tif]
