# Supplementary material for: A phase-shifting anterior-posterior network organizes global phase relations
Source: PLoS One. 2024 Feb 12;19(2):e0296827. doi: 10.1371/journal.pone.0296827 (PMC10861041; doi:10.1371/journal.pone.0296827)
Supplement: Attachment — Submitted filename: MencelogluEtAl_Response to Reviews_R2.pdf [file pone.0296827.s003.pdf]

Satoru Suzuki
Professor

Department of Psychology and Interdepartmental Neuroscience Program
2029 Sheridan Road
Evanston, IL 60208-2710 USA

Telephone: (847) 467-1271
Fax: (847) 491-7859
Email: satoru@northwestern.edu

NORTHWESTERN UNIVERSITY

10/9/2022

Dear Academic Editor:

We are submitting a second revision of our manuscript titled, "A phase-shifting anterior-posterior network organizes global phase relation" for consideration for publication in *PLOS ONE*.

As this submission is the result of our appeal of the editorial decision made to our R1 submission, (1) our letter of appeal, (2) the action letter for our original submission, (3) our point-by-point response to the reviewer comments for the original submission, and (4) the action letter and Reviewer 2's comments on our R1 submission (no comments from Reviewer 1), are provided below. Our detailed response to Reviewer 2's comments on R1 is included in the appeal letter (1).

In the track-change version of the R2 manuscript, the revisions made based on the initial reviewer comments are highlighted in red text, and the revisions made based on Reviewer 2's comments on R1 are highlighted in blue text.

Thank you for your consideration of our manuscript.

Sincerely,

Satoru Suzuki

(1) Our letter of appeal

Dear PLOS ONE team:

We would like to appeal the decision made by the action editor Stavros I. Dimitriadis on our REVISED manuscript titled, a phase-shifting anterior-posterior network organizes global phase relations. What we feel most troubling is the gross inconsistency in the review process. Dimitriadis, Reviewer 1, and Reviewer 2 all expressed enthusiasm for our original manuscript with BOTH reviewers responding to the first review criterion as “partly” and responding “yes” to all other review criteria. Reviewer 1 provided extensive comments to which we carefully responded point by point. Both reviewers voluntarily commented that the manuscript was “well written.” Reviewer 2 only gave general suggestions, (1) to make the paper shorter if possible, (2) to make the figures higher-resolution (which we did in the revision), and (3) to comment on the extent of volume-conduction effects potentially affecting our results (which we did in the revision).

We just received a rejection notice with no comments from Reviewer 1. We assume that Reviewer 1, who provided extensive and thoughtful comments to our original submission, was satisfied with our response/revision. What is problematic is that Reviewer 2 haphazardly changed his/her mind from his/her first review. Although he/she responded “yes” to all review criteria except responding “partly” to the first criterion in the original submission, he/she suddenly responded “no” to all review criteria in the revision despite the fact that none of the results or statistical analyses changed. In particular, as we made the raw data publically available at the time of the original submission, suddenly responding “no” to that criterion for the revision is blatantly neglectful.

Even worse, Reviewer 2 does NOT provide any logical explanations for his/her change of mind in the comments section of the review. Now, he/she says that the results are difficult to interpret while he/she said in his/her original review that, although the manuscript was long it was **well written** and that the results were evident from the figures; we emphasize that Reviewer 1 also voluntarily commented that the manuscript was “**well written**.”

The only objective comments Reviewer 2 provided for the revision regarded (1) our careless citation of BESA in our general discussion of commonly used EEG source-imaging methods and (2) the extent of volume-conduction effects potentially affecting our study. Reviewer 2 is correct that we miscited BESA; it is a software package that researchers may use to run various EEG source-imaging analyses so that it is meaningless to mention BESA along with sLORETA and Beamformer (which are source-imaging methods that can be applied using the BESA package). We corrected this mistake in the attached version (see blue text; red text indicates the revisions we made to the original manuscript). We appreciate Reviewer 2 for detecting the mistake, but this was something that we added in the revision upon Reviewer 2’s request to provide some discussion of commonly used source-imaging methods; it has zero impact on the quality or validity of our results.

Regarding the extent of volume-conduction effects in our study, we explained that we reduced them using the surface-Laplacian transform with references to articles stating that the surface-Laplacian transform (of the type we used applied to the electrode configuration we used) reduces volume conduction effects to 1-3 cm. While Reviewer 2 may disagree with the cited literature, we directly confirmed that our study indeed achieved this level of spatial resolution and presented the evidence in the revised Figure 4. This should be decisive.

We have dealt with countless journal submissions and reviews (also recently published several papers with PLOS ONE), but this was **by far the most inconsistent and irresponsible review process** we have ever experienced. We would like to appeal the decision because we believe that anyone who reads the comments we received from Dimitriadis and both reviewers on our original submission, our point-by-point response to those comments, and the Reviewer 2's perplexingly discrepant comments on the revision, would see that Reviewer 2's behavior is unacceptably inconsistent, and Dimitriadis irresponsibly endorsed this behavior.

We hope that you would be willing to form an appropriate committee (not including Dimitriadis or Reviewer 2) to evaluate the reviews we received on our original submission, our responses to their comments, our revision, and Reviewer 2's brief and inconsistent review on the revision (please see below). We would also like to remind you again that Reviewer 1, who provided many thoughtful comments to our original submission, accepted our revision (we assume). We would be more than willing to make extensive revisions and/or run new experiments/analyses if the committee detected any meaningful scientific deficits in our study. However, we would not be compelled to revise our paper based on the unacceptably inconsistent and haphazard comments given to us by Reviewer 2. Rigorous reviews improve science, but inconsistent/haphazard reviews hurt science. We are extremely disappointed that we received the latter from PLOS ONE.

We have been selectively submitting our research to PLOS ONE because we value the journal's mission of promoting rigorous science without subjective biases or preferences. In this regard, it is disturbing that Dimitriadis is asking us to reduce the scope of our study based on Reviewer 2's subjective preference, that was not shared by Reviewer 1 or Dimitriadis in the initial review.

Our paper is entirely focused on supporting our claim that phase-shifting anterior-posterior long-distance network controls the dynamics of modularity in EEG phase relations. The paper is not unusually long. It is logically laid out to present layers of converging evidence step by step. We reiterate that BOTH reviewers evaluated the original submission to be "well written" and Dimitriadis agreed with them; neither the paper layout nor the amount of data presented changed in the revision. All data figures are necessary to convincingly support our claim. Compelling us to provide less evidence to make the paper shorter and more stylish is **contrary to practicing rigorous science**. We also emphasize that the decision to reject the revision has **NO basis on scientific concerns**. We believe an editorial decision like this severely undermines PLOS ONE's stated mission of promoting rigorous science without subjective biases/preferences.

Thank you for your time and consideration. However, if you determine that it will take months to make your decision, please let us know so that we may submit our paper elsewhere.

Below, please see the action letter and reviews for the original submission, our point-by-point response to the reviews, and the action letter and reviews for the revision.

Sincerely,

Satoru Suzuki

(2) The action letter for our original submission

PONE-D-22-00971
A phase-shifting anterior-posterior network organizes global phase relations
PLOS ONE

Dear Dr. Suzuki,

Thank you for submitting your manuscript to PLOS ONE. After careful consideration, we feel that it has merit but does not fully meet PLOS ONE's publication criteria as it currently stands. Therefore, we invite you to submit a revised version of the manuscript that addresses the points raised during the review process.

Your draft has been evaluated by the two reviewers. Both of them including myself found the article very interesting and informative for the field.
The comments raised by the two reviewers can be addressed if you wish to revise the manuscript.

Please submit your revised manuscript by Jun 05 2022 11:59PM. If you will need more time than this to complete your revisions, please reply to this message or contact the journal office at plosone@plos.org. When you're ready to submit your revision, log on to https://www.editorialmanager.com/pone/ and select the 'Submissions Needing Revision' folder to locate your manuscript file.

We look forward to receiving your revised manuscript.

Kind regards,

Stavros I. Dimitriadis
Academic Editor
PLOS ONE

Comments to the Author

1. Is the manuscript technically sound, and do the data support the conclusions?

The manuscript must describe a technically sound piece of scientific research with data that supports the conclusions. Experiments must have been conducted rigorously, with appropriate controls, replication, and sample sizes. The conclusions must be drawn appropriately based on the data presented.

Reviewer #1: Partly

Reviewer #2: Partly

2. Has the statistical analysis been performed appropriately and rigorously?

Reviewer #1: Yes

Reviewer #2: Yes

3. Have the authors made all data underlying the findings in their manuscript fully available?

The PLOS Data policy requires authors to make all data underlying the findings described in their manuscript fully available without restriction, with rare exception (please refer to the Data Availability Statement in the manuscript PDF file). The data should be provided as part of the manuscript or its supporting information, or deposited to a public repository. For example, in addition to summary statistics, the data points behind means, medians and variance measures should be available. If there are restrictions on publicly sharing data—e.g. participant privacy or use of data from a third party—those must be specified.

Reviewer #1: Yes

Reviewer #2: Yes

4. Is the manuscript presented in an intelligible fashion and written in standard English?

PLOS ONE does not copyedit accepted manuscripts, so the language in submitted articles must be clear, correct, and unambiguous. Any typographical or grammatical errors should be corrected at revision, so please note any specific errors here.

Reviewer #1: Yes

Reviewer #2: Yes

(3) Our point-by-point response to the reviewer comments for the original submission

Dear Dr. Dimitriadis:

Thank you for obtaining two expert reviews and for taking the time to read the manuscript yourself. The reviewers' comments were very helpful, and have made us critically think about some important issues related to the analyses of inter-site phase relations.

We believe that we have addressed all the reviewers' comments in our Response to Reviewers' Comments below and in the revised manuscript (all substantive changes are highlighted in red). If you and/or either of the reviewers feel that we did not appropriately address any of their concerns, we apologize and ask that they would be so kind as to explain to us how we came up short in addressing their concerns.

Thank you also for volunteering your time to be an academic editor for *PLOS ONE.* We have published our work in a variety of journals, but *PLOS ONE* is our current go-to outlet as we strongly appreciate their support of rigorous science independent of subjective preferences for topics and methods.

Sincerely,

Satoru Suzuki

Response to Reviewers' Comments:

Reviewer 1:

The importance of phase-difference distribution (and of the single values associated with specific pairs of regions) is undubt. The authors investigated global phase-differences to understand the general context in a large cohort of EEG subject, and they found very interesting results. The manuscript is also well written. Nevertheless, I found a few (potential) issues in analysis (1-the fact that it is a scalp level, instead of being at source level, 2-the fact that the phase-differences are computed as the differences between separately calculated single phases, instead of directly computing the phase-difference e.g. from cross-spectral quantities, and 3-the fact that an orthogonalization-procedure to avoid artificial instantaneous coupling has not been applied, thus generating distributions with most of the mass concentrated close to 0 and pi). Prior to publish the manuscript, I would thus suggest a major revision.

We thank the reviewer for these summary comments. Please see our response to each of his/her thoughtful comments below.

Introduction: the whole section (except for the first lines) does not seem to be an introduction of a paper but a synthesis of the methods, results and discussion sections together; I suggest the

authors to reshape the introduction in order to focus on the reasons the justified this manuscript. Note: the reasons are somehow there already, but the whole paper would benefit from a deepening into the modern literature, e.g. are there studies focusing on lag/phase-difference distributions? Did the authors of the previously published studies investigate all the frequency bands? Are there other limits in those research papers? Instead just a few articles have been cited so far.

Based on our literature survey, most studies focused on identifying functional networks based on inter-regional connectivity, measured as the strengths of phase locking around $\pi/2$ inferred with the imaginary component of PLV (phase locking value), PLI (phase lag index), orthogonalization methods (please see below), etc. Studies that examined phase lags tended to focus on inferring directional connectivity between specific regions using measures such as phase-slope index and phase-transfer entropy. We were unable to find any prior studies that systematically examined global topological distributions of phase relations. We did find a recent computational study that examined whole-brain phase relations using a network model built with white-matter connectivity inferred from the connectome data; this study, which also found the clustering of phase relations around 0 and π, is now cited (please see below). We have augmented the introduction section to make these points. We also cite a recent review paper on the use of phase locking measures to identify functional networks as well as studies that comprehensively compared phase-locking based functional networks with networks inferred with fMRI BOLD correlations and DTI.

Methods: I would suggest to perform the analysis at source level (i.e. after the application of an inverse method able to estimate source activity from channel level data) in order to further reduce possible artifactual confounds and to better and directly characterise the spatial features at cortical level. If the authors do not consider the application of a source level analysis necessary, they might at least discuss why the applied scalp level analysis does not suffer from usual confounds.

We agree that it is necessary to reduce volume-conduction effects using some method of source reconstruction before conducting analyses on phase coupling (e.g., Cohen, 2014). The Surface-Laplacian (SL) transform applied to scalp-recorded EEG data (theoretically) estimates the spatial distribution of macroscopic current sources/sinks on the dura surface. Nunez et al. (1994) showed that the SL transform produced similar dura sources to those inferred with a volume-conduction model that deconvolved surface potentials using a model of thicknesses and impedances of scalp and skull. Commonly used inverse source-modeling methods such as non-adaptive methods including BESA and sLORETA (reviewed in Tenke & Kayser, 2012), and adaptive methods such as Beamforming (Cohen et al., 2015) approximated the simulated sources and/or extracted neural correlates of behaviors to a similar degree as the SL transform. Source modeling with individualized brain models constrained by MRI and fMRI has been shown to offer superior spatial resolution (Cottereau et al., 2015), though this option is not available for the current study. To our knowledge, there is no consistent evidence to suggest that popular source-reconstruction methods such as BESA, sLORETA, Beamforming, etc. reduce volume conduction effects to a greater degree than the SL transform. Our preference thus is to use the SL transform because it is the most general source-imaging method that relies the least on model-specific assumptions and free parameters. We acknowledge that Reviewer 1 and/or Reviewer 2 may still prefer the use of one of the popular inverse source models. Nevertheless, we believe that this would be a matter of personal preference rather than scientific rigor. As we will make both raw and the SL-transformed EEG data publicly available,

interested researchers could compare our results with those generated with the various inverse source-modeling methods.

As suggested by Reviewer 1, we now include a brief discussion of why we chose to use the SL transform as our method of source imaging. We have also strengthened Figure 4 to clearly indicate the rapid attenuation of PCV as a function of inter-site distance. We now present the attenuation slopes for four equal-distance intervals, 0-0.5 (greater than the majority of neighbor distances; M = 0.42, SD = 0.085), 0.5-1, 1-1.5, and 1.5-2. The slopes for the first interval were much steeper (typically by more than an order of magnitude) than the slopes for the rest of the intervals, confirming that a substantial portion of volume-conduction effects attenuated within the distances of neighboring sites after the SL transformation (consistent with Cohen, 2014, and Tenke & Kayser, 2012).

Reviewer 1 noted the prevalence of low (near 0) and high (near π) phase relations ($|\Delta\theta|$) in our results. Some of those may reflect volume-conduction effects and current-source-and-sink pairs, respectively, especially at short distances, as we acknowledge in our manuscript. Nevertheless, we now cite a computational study that used a whole-brain network built on white-matter connectivity to simulate global distributions of phase relations. Consistent with our results, the simulation yielded distributions that were clustered around in-phase (0) and anti-phase (π) relations (Petkoski et al., 2018).

Cohen, M. X. (2014). *Analyzing Neural Time Series Data: Theory and Practice*, MIT Press.

Cohen, M. X. (2015). Comparison of different spatial transformations applied to EEG data: A case study of error processing. *International Journal of Psychophysiology, 97*, 245-257.

Cottereau, B. R., Ales, J. M., & Norcia, A. M. (2015). How to use fMRI functional localizers to improve EEG/MEG source estimation. *Journal of Neuroscience Methods, 250*, 64-73.

Nunez, P. L., Silbersten, R. B., Cadusch, P. J., Wijesinghe, R. S., Westdorp, A. F., & Srinivasan, R. (1994). A theoretical and experimental study of high resolution EEG based on surface Laplacians and cortical imaging. *Electroencephalography and Clinical Neurophysiology, 90*, 40-57.

Petkoski, S., Palva, J. M., Jirsa, V. K. (2018). Phase-lags in large scale brain synchronization: methodological considerations and in-silico analysis. *PLOS Computational Biology, 14(7)*: e1006160.

Tenke, C. E., & Kayser, J. (2012). Generator localization by current source density (CSD): Implications of volume conduction and field closure at intracranial and scalp resolutions. *Clinical Neurophysiology, 123*, 2328-2345.

Methods: were the phase-differences calculated as the differences between the computed single phases? I suggest to directly compute the phase-difference instead of separately computing the two single phases to be subtracted (this is an issue that led a lot of researchers to compute the phase-difference from the cross-spectrum and not from the two single Fourier-phases).

We very much appreciate this comment. If we computed inter-site phase differences inappropriately, our results would be meaningless. If we understood Reviewer 1's comment

correctly, we believe what we did was in fact what Reviewer 1 suggests we should have done. Cross-spectral density can be computed with various time-frequency decomposition methods. We chose Morlet wavelets as they are (to our knowledge) most effective at dissociating the phase and amplitude of sinusoidal components for signals that contain many such components. Let $W_1(t,f)$ and $W_2(t,f)$ be the time series of complex wavelet-convolved signals from site 1 and site 2, respectively. The time series of cross-spectral density between these sites can be computed as, $S_{12}(t,f) = W_2(t,f)^*W_1(t,f)$ or $S_{21}(t,f) = W_1(t,f)^*W_2(t,f)$, where * indicates complex conjugation, t is time, and f is wavelet center frequency. Because $W_1 = |W_1|e^{i\phi 1}$ and $W_2 = |W_2|e^{i\phi 2}$ owing to Euler's formula, where ϕ's are the phase angles and |W|'s are the amplitudes of the complex signals, we have $S_{12} = |W_1||W_2|e^{i(\phi 1-\phi 2)}$ or $S_{21} = |W_2||W_1|e^{i(\phi 2-\phi 1)}$. Thus, the complex phase difference between site 1 and site 2 can be computed as, $\mathbf{e^{i(\phi 1-\phi 2)} = S_{12}/(|W_1||W_2|)}$ **or** $\mathbf{e^{i(\phi 2-\phi 1)} = S_{21}/(|W_2||W_1|)}$**—Equation 1**. We believe this is what Reviewer 1 suggests and that is exactly what we did. Incidentally, this procedure is mathematically equivalent to computing phase angles separately for W_1, $\phi 1$, and W_2, $\phi 2$, and taking their difference in the complex representation, $e^{i(\phi 1-\phi 2)}$ or $e^{i(\phi 2-\phi 1)}$. To improve clarity, we now provide the mathematical formula (Equation 1) that we used to extract time series of complex inter-site phase differences.

Methods: by looking at the |deltatheta|-distributions, it seems that the mass of the (magnitude of the) phase differences is basically either close to 0 or close to pi (U-shaped). This feature may reflect the fact that volume conduction is still playing a confounding role. I suggest to regress-out the contribution of one signal from the second signal before calculating the phase-difference; this process is known as "orthogonalization" and it is also applied to phase-based (but not robust to volume conduction) connectivity metrics. If the authors do not believe this could be the case, they may just show (as examples) that using the orthogonalization does not change the phase-difference distribution for a few site-pairs (or explain that it can introduce additional confound, instead of improving the analysis).

Our knowledge of "orthogonalization" in the context of EEG/MEG connectivity analyses is based on Hipp et al. (2012) and Brookes et al. (2012). Orthogonalization procedures can be applied to time-frequency convolved complex signals obtained with wavelet transform, Hilbert transform, etc. As above, we let $W_1(t,f)$ and $W_2(t,f)$ represent the convolved complex signals from site 1 and site 2, respectively, as a function of time (t) and frequency (f). Hipp et al. defines W_2 orthogonalized to W_1 as $\mathbf{Im(W_1{}^*W_2/|W_1|)}$ (Im is to take the imaginary part), which equals $|W_2|\sin(\phi 12)$, that is the magnitude of the component of W_2 in the direction perpendicular to W_1 in the complex plane (the length of the black arrow in the figure). Brookes et al. defines W_2 orthogonalized to W_1 as $\mathbf{W_2 - Re(W_1{}^+W_2)W_1}$ (Re is to take the real part and + indicates Moore-Penrose inverse), which gives the component of W_2 in the direction perpendicular to W_1 expressed as a vector in the complex plane (the black arrow in the figure). The magnitude of this vector is given by Hipp et al.'s formula; thus, Hipp et al.'s and Brookes et al.'s orthogonalization methods are mathematically equivalent. Either method provides the orthogonalized site 2 signals (relative to site 1 signals) reflecting the amplitude of site 2 complex signals projected to the $\pi/2$ rotated axis relative to site 1 complex signals, thus systematically discounting phase differences closer to

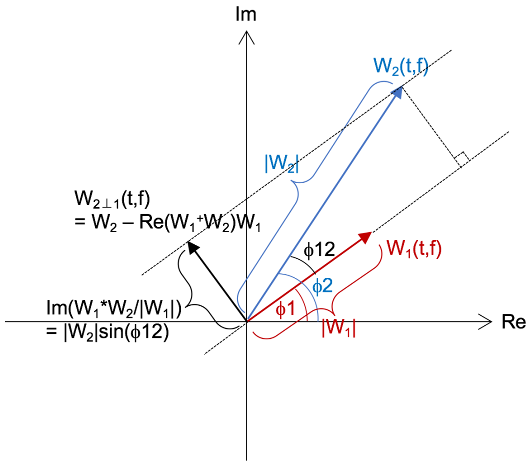

0 or π (see the figure). Thus, these orthogonalization procedures yield the strength of pairwise phase relations away from 0 or π, by systematically reducing the contributions from phase relations close to 0 or π and eliminating the contributions from phase relations at 0 or π. As the reviewer points out, a similar outcome can be obtained by residualizing the signals from one site to its linear relationship with the signals from another site, given that the signals are appropriately band-pass filtered at each frequency (Brookes et al., 2012).

We agree that this type of orthogonalization strategy is useful when the goal is to evaluate the strength of pairwise phase locking regardless of phase differences. In that case, it makes good sense to reduce/eliminate contributions from phase differences at or near 0 or π which could reflect volume-conduction effects. However, our goal was different. We wanted to systematically examine the topological distributions of pairwise phase relations, where those close to or at 0 or π are just as relevant as those away from 0 or pi. Hipp et al's and Brookes et al.'s orthogonalization procedure necessarily forces the pairwise phase relations between the site 2 signals orthogonalized to site 1 signals and site 1 signals to be $\pi/2$ apart (because the procedure uses vector orthogonalization in the complex plane; please see the figure).

There are many procedures such as orthogonalization (discussed above), imaginary coherence, and phase-lag index, etc. to discount/remove phase locking at 0 and π (which may partially reflect volume-conduction effects) for phase-based connectivity analyses. However, there are no effective procedures (to our knowledge) to subtract out volume-conduction effects while estimating phase relations. This is difficult (likely impossible) because it would require precise knowledge of the amplitude and phase of volume-conducted contributions. If the estimates were inaccurate, an attempt to subtract out the volume-conducted component (as vectors in the complex plane) would artifactually alter phase relations in unpredictable directions. In our analysis, we used the SL transform to reduce volume-conduction effects, and beyond that, we at least know that any volume-conduction effects would bias the phase relations to be closer to 0 or π than their true values. That said, we are aware that our knowledge may well be limited. Reviewer 1 may be familiar with an orthogonalization procedure fundamentally different than those used by Hipp et al. and Brookes et al., that would remove volume-conduction effects while preserving "true" phase relations at 0 and π. If so, if Reviewer 1 would be so kind as to share the relevant papers with us, we would carefully study the method and apply it to the current study if appropriate.

Brookes et al. (2012). Measuring functional connectivity in MEG: A multivariate approach insensitive to linear source leakage. *NeuroImage, 63(2)*, 910-920.

Hipp et al. (2012). Large-scale cortical correlation structure of spontaneous oscillatory activity. *Nature Neuroscience, 15*, 884-890.

Reviewer 2:

In the present manuscript, the authors present a quite interesting approach for modeling phase shifting among anterior - posterior sensor based networks using phase modularity for different individual frequencies. The manuscript is well-written however quite long making it difficult to focus and understand the results. This is more than clear from the figures even though they present interesting results. In this respect, the authors need to take care the figure visibility because it is difficult to make clear interpretations based on the low quality of the figures.

We thank Reviewer 2 for the summary comments. We understand the length of the manuscript, but we were unable to find things to cut. In response to Reviewer 1's comments, we have made improvements on the accuracy of our descriptions. We hope that those improvements increased the overall clarity of presentation despite the length. We have increased the resolution of the figures.

One question is how the authors verify that the presented connectivity results can be supported by source analysis based phase modularity since it is not entirely clear how the volume conduction effects are suppressed. What about the inter-subject variability of skull conductivity for which bone is quite thick at the anterior and posterior regions.

As Reviewer 1 also brought up this issue, we here copy our response to Reviewer 1.

We agree that it is necessary to reduce volume-conduction effects using some method of source reconstruction before conducting analyses on phase coupling (e.g., Cohen, 2014). The Surface-Laplacian (SL) transform applied to scalp-recorded EEG data (theoretically) estimates the spatial distribution of macroscopic current sources/sinks on the dura surface. Nunez et al. (1994) showed that the SL transform produced similar dura sources to those inferred with a volume-conduction model that deconvolved surface potentials using a model of thicknesses and impedances of scalp and skull. Commonly used inverse source-modeling methods such as non-adaptive methods including BESA and sLORETA (reviewed in Tenke & Kayser, 2012), and adaptive methods such as Beamforming (Cohen et al., 2015) approximated the simulated sources and/or extracted neural correlates of behaviors to a similar degree as the SL transform. Source modeling with individualized brain models constrained by MRI and fMRI has been shown to offer superior spatial resolution (Cottereau et al., 2015), though this option is not available for the current study. To our knowledge, there is no consistent evidence to suggest that popular source-reconstruction methods such as BESA, sLORETA, Beamforming, etc. reduce volume conduction effects to a greater degree than the SL transform. Our preference thus is to use the SL transform because it is the most general source-imaging method that relies the least on model-specific assumptions and free parameters. We acknowledge that Reviewer 1 and/or Reviewer 2 may still prefer the use of one of the popular inverse source models. Nevertheless, we believe that this would be a matter of personal preference rather than scientific rigor. As we will make both raw and the SL-transformed EEG data publicly available, interested researchers could compare our results with those generated with the various inverse source modeling methods.

As suggested by Reviewer 1, we now include a brief discussion of why we chose to use the SL transform as our method of source imaging. We have also strengthened Figure 4 to clearly indicate the rapid attenuation of PCV as a function of inter-site distance. We now present the attenuation slopes for four equal-distance intervals, 0-0.5 (greater than the majority of neighbor distances; M = 0.42, SD = 0.085), 0.5-1, 1-1.5, and 1.5-2. The slopes for the first interval were much steeper (typically by more than an order of magnitude) than the slopes for the rest of the intervals, confirming that a substantial portion of volume-conduction effects attenuated within the distances of neighboring sites after SL transformation (consistent with Cohen, 2014, and Tenke & Kayser, 2012).

Reviewer 1 noted the prevalence of low (near 0) and high (near π) phase relations ($|\Delta\theta|$) in our results. Some of those may reflect volume-conduction effects and current-source-and-sink pairs, respectively, especially at short distances, as we acknowledge in our manuscript. Nevertheless, we now cite a computational study that used a whole-brain network built on white-matter

connectivity to simulate distributions of phase relations. Consistent with our results, the simulation yielded distributions that were clustered around in-phase (0) and anti-phase (π) relations (Petkoski et al., 2018).

Cohen, M. X. (2014). *Analyzing Neural Time Series Data: Theory and Practice*, MIT Press.

Cohen, M. X. (2015). Comparison of different spatial transformations applied to EEG data: A case study of error processing. *International Journal of Psychophysiology, 97*, 245-257.

Cottereau, B. R., Ales, J. M., & Norcia, A. M. (2015). How to use fMRI functional localizers to improve EEG/MEG source estimation. *Journal of Neuroscience Methods, 250*, 64-73.

Nunez, P. L., Silbersten, R. B., Cadusch, P. J., Wijesinghe, R. S., Westdorp, A. F., & Srinivasan, R. (1994). A theoretical and experimental study of high resolution EEG based on surface Laplacians and cortical imaging. *Electroencephalography and Clinical Neurophysiology, 90*, 40-57.

Petkoski, S., Palva, J. M., Jirsa, V. K. (2018). Phase-lags in large scale brain synchronization: methodological considerations and in-silico analysis. *PLOS Computational Biology, 14(7)*: e1006160.

Tenke, C. E., & Kayser, J. (2012). Generator localization by current source density (CSD): Implications of volume conduction and field closure at intracranial and scalp resolutions. *Clinical Neurophysiology, 123*, 2328-2345.

(4) The action letter and Reviewer 2's comments on our R1 submission (no comments from Reviewer 1)

PONE-D-22-00971R1
A phase-shifting anterior-posterior network organizes global phase relations
PLOS ONE

Dear Dr. Suzuki,

Thank you for submitting your manuscript to PLOS ONE. After careful consideration, we have decided that your manuscript does not meet our criteria for publication and must therefore be rejected.

Specifically:

Dear authors

I have read carefully your paper, and I regret to inform you that it is not appropriate for publication.
I totally agree with the comments raised by the reviewer.
You have to narrow down your findings into a specific direction, and also to reduce the size of the draft.
Secondly, you must be careful when you use the term 'diminish' the volume conduction effects, forward problem,
the use of BESA etc.
I definitely recommend to resubmit the work after carefully addressing the suggestions.

I am sorry that we cannot be more positive on this occasion, but hope that you appreciate the reasons for this decision.

Kind regards,

Stavros I. Dimitriadis
Academic Editor
PLOS ONE

Comments to the Author

1. If the authors have adequately addressed your comments raised in a previous round of review and you feel that this manuscript is now acceptable for publication, you may indicate that here to bypass the “Comments to the Author” section, enter your conflict of interest statement in the “Confidential to Editor” section, and submit your "Accept" recommendation.

Reviewer #2: (No Response)

2. Is the manuscript technically sound, and do the data support the conclusions?

The manuscript must describe a technically sound piece of scientific research with data that supports the conclusions. Experiments must have been conducted rigorously, with appropriate controls, replication, and sample sizes. The conclusions must be drawn appropriately based on the data presented.

Reviewer #2: No

3. Has the statistical analysis been performed appropriately and rigorously?

Reviewer #2: No

4. Have the authors made all data underlying the findings in their manuscript fully available?

The PLOS Data policy requires authors to make all data underlying the findings described in their manuscript fully available without restriction, with rare exception (please refer to the Data Availability Statement in the manuscript PDF file). The data should be provided as part of the manuscript or its supporting information, or deposited to a public repository. For example, in addition to summary statistics, the data points behind means, medians and variance measures should be available. If there are restrictions on publicly sharing data—e.g. participant privacy or use of data from a third party—those must be specified.

Reviewer #2: No

5. Is the manuscript presented in an intelligible fashion and written in standard English?

PLOS ONE does not copyedit accepted manuscripts, so the language in submitted articles must be clear, correct, and unambiguous. Any typographical or grammatical errors should be corrected at revision, so please note any specific errors here.

Reviewer #2: No

Reviewer #2: The results are difficult to interpret. The reader gets lost in the long and difficult-to-read+interpret results. Also, the authors misuse terms like BESA / sLoreta (one is a commercial tool for source analysis while the other is an inverse approach for reconstructing brain activity. None of sLoreta or BESA can reduce the effects of volume conduction. Actually such sentences does not make sense. The correct here is to talk about forward problem and how the authors deal with it. In any case the main reason of rejecting this work is the large amount of results (Fig. 4, 5, 6, 7, 10) that lead to confusion and making the article very long and difficult to understand. Comments for improvements are to decide which are the most distinctive results, describe them well and consistently in a manner that the authors will attract easily many readers. Take this opportunity and if possible resubmit the work in the same journal.
