# Supplementary material for: A phase-shifting anterior-posterior network organizes global phase relations
Source: PLoS One. 2024 Feb 12;19(2):e0296827. doi: 10.1371/journal.pone.0296827 (PMC10861041; doi:10.1371/journal.pone.0296827)
Supplement: Attachment — Submitted filename: MencelogluEtAl_PhaseModularity_R3_response.pdf [file pone.0296827.s006.pdf]

Our response to each of the points raised by Reviewers 2, 3, 4, and 5.
We pasted each of the reviewers' comments in italics, immediately followed by our response in blue text.

Reviewer #2*: Sorry but the review really disagrees with the authors response, the manuscript by the view of the results / figures really de-motivates any try for real scientific review. It is very difficult to understand and interpret them. Please revise your work making it readable in terms of figure and maybe the reviewer will be willing to really review the work."*

Our response: We appreciate that Reviewer 2 took his/her time to look at our manuscript. We also respect his/her personal opinion that our manuscript is difficult to comprehend. However, we respectfully disagree with the assessment. We believe that given the nature of the current research, the structure of the writing and the amount of data presented are appropriate and the other reviewers broadly agree with our assessment. We cannot think of any way to simplify the manuscript without compromising scientific rigor.

Reviewer #3: *Manuscript Number: PONE-D-22-00971R2*
Manuscript Title: A phase-shifting anterior-posterior network organizes global phase relations
Comments/ Review:
The paper is well written and clear illustrations are provided.
The manuscript presents interesting and novel approach for modelling phase shifting among interiors. The method and material section is enough detailed to check the suitability skilled investigators. The paper has ample and both old & latest references are mentioned and should be accepted for the journal.

Our response: We thank Reviewer 3 for carefully reading our manuscript and providing his/her thoughtful assessment of it.

Reviewer #4: *Manuscript Number: PONE-D-22-00971R2*
The research proposed A phase-shifting anterior-posterior network to organize global phase relations, and the author conducted a detailed analysis and discussion.
The anterior-posterior network likely plays a fundamental functional role as it controls phase modularity across various frequencies and behavioral conditions.
These results may motivate future investigations into the functional roles of phase modularity as well as the anterior-posterior network that controls it.

1. It is suggested that the paper should be written in a standard way, and the chart should be placed in the text, which will increase the readability of the paper.

Our response: We appreciate this comment. However, our manuscript is written "in a standard way" as it contains the introduction, methods, results, and discussion sections in that order. Perhaps, Reviewer 4 may consider our manuscript to be non-standard because it presents observational/discovery science, seeking to discover general rules governing global neural dynamics, rather than the typical hypothesis-testing science where hypotheses are stated at the beginning and are evaluated in the results section. Although some journals may be reluctant to publish observational/discovery science, on the basis of their mission statement, *PLOS ONE* is not one of them.

Regarding the recommendation to place the chart in the text, it would be helpful if Reviewer 4 would clarify what he/she refers to as a chart which can refer to a table, a graph, or a diagram. We understand that it is customary to present tables separately from figures, but we do not present any tables.

2. Research methods should be elaborated in further detail so that the researcher can replicate the work.

Our response: We appreciate this concern as it is important to provide sufficient methodological details so that interested scientists will be able to replicate our study. We have included additional information on data collection, namely the period in which we recruited our participants and our exclusion criteria (no recruited participants were excluded). Further, given the comments from Reviewer 5, we now provide the MATLAB code that we used to compute the modularity index, *q*, as a supplementary material. We hope that with these additions Reviewer 4 will agree that we now provide sufficient methodological details.

3. There are some papers that may be helpful to improve the quality of this manuscript if they can be added to the reference. https://doi.org/10.1088/1741-2552/acbe20 https://doi.org/10.1016/j.patcog.2021.107885 https://doi.org/10.3389/fpsyt.2021.837149 and https://doi.org/10.3389/fncom.2022.942979

Our response: We appreciate that Reviewer 4 brought to our attention these interesting papers. However, these are all about EEG pattern classification which is not directly relevant to the focus of our study. We would also like to respectfully point out that at our institution, it is considered unethical for a reviewer to encourage authors to cite specific papers without articulating their direct relevance. As we are bound by our institution's code of conduct, we are unable to consider this recommendation without further justification.

Reviewer #5: *In the present manuscript, the authors investigate phase differences between EEG sites during different behavioral conditions: resting with the eyes closed and watching a silent nature video.*
They analyze spontaneous fluctuations in phase modularity and compare the spatial distributions of phase relations between states of maximal and minimal modularity.
Even though there are many different ways to preprocess EEG data, their methods are well explained and suitable, especially for the sensor level.
The results are potentially interesting for the computational and experimental neuroscience communities. I would only suggest a more detailed explanation about how they measure the phase modularity instead of only mentioning references 14 and 21. Therefore, I recommend it for publication.

Our response: We thank Reviewer 5 for his/her thoughtful comments. Instead of regurgitating the detailed descriptions of *q* from the articles that we cite, we now provide the MATLAB code used to compute *q* as a supplementary material (modularity_und.m). We hope that Reviewer 5 will find this to be satisfactory.
