# Supplementary material for: A phase-shifting anterior-posterior network organizes global phase relations
Source: PLoS One. 2024 Feb 12;19(2):e0296827. doi: 10.1371/journal.pone.0296827 (PMC10861041; doi:10.1371/journal.pone.0296827)
Supplement: Attachment — Submitted filename: Recommendations.docx [file pone.0296827.s004.docx]

**Manuscript Number:** PONE-D-22-00971R2
**Manuscript Title:** A phase-shifting anterior-posterior network organizes global phase relations

**Comments/ Review:**

The paper is well written and clear illustrations are provided.

The manuscript presents interesting and novel approach for modelling phase shifting among interiors.

The method and material section is enough detailed to check the suitability skilled investigators.

The paper has ample and both old & latest references are mentioned and should be accepted for the journal.
