# Supplementary material for: A phase-shifting anterior-posterior network organizes global phase relations
Source: PLoS One. 2024 Feb 12;19(2):e0296827. doi: 10.1371/journal.pone.0296827 (PMC10861041; doi:10.1371/journal.pone.0296827)
Supplement: Attachment — Submitted filename: renamed_7d8bd.docx [file pone.0296827.s005.docx]

The research proposed A phase-shifting anterior-posterior network to organize global phase relations, and the author conducted a detailed analysis and discussion.

The anterior-posterior network likely plays a fundamental functional role as it controls phase modularity across various frequencies and behavioral conditions.

These results may motivate future investigations into the functional roles of phase modularity as well as the anterior-posterior network that controls it.

1. It is suggested that the paper should be written in a standard way, and the chart should be placed in the text, which will increase the readability of the paper.
2. Research methods should be elaborated in further detail so that the researcher can replicate the work.
3. There are some papers that may be helpful to improve the quality of this manuscript if they can be added to the reference. <https://doi.org/10.1088/1741-2552/acbe20> <https://doi.org/10.1016/j.patcog.2021.107885> <https://doi.org/10.3389/fpsyt.2021.837149> and https://doi.org/[10.3389/fncom.2022.942979](https://doi.org/10.3389%2Ffncom.2022.942979)
